# Comparison of novel and existing methods for detecting differentially methylated regions

**DOI:** 10.1186/s12863-018-0637-4

**Published:** 2018-09-17

**Authors:** Samantha Lent, Hanfei Xu, Lan Wang, Zhe Wang, Chloé Sarnowski, Marie-France Hivert, Josée Dupuis

**Affiliations:** 1 Department of Biostatistics, Boston University School of Public Health, 801 Massachusetts Avenue, 3rd Floor, Boston, MA 02118 USA; 2 Bioinformatics Program, Boston University, 44 Cummington Mall, Boston, MA 02215 USA; 3 Obesity Prevention Program, Department of Population Medicine, Harvard Medical School and Harvard Pilgrim Health Care Institute, 401 Park Drive, Suite 401 East, Boston, MA 02215 USA; 4 Diabetes Unit, Massachusetts General Hospital, 50 Staniford Street, Suite 340, Boston, MA 02144 USA

**Keywords:** Epigenetics, DNA methylation, Differentially methylated regions

## Abstract

**Background:**

Single-probe analyses in epigenome-wide association studies (EWAS) have identified associations between DNA methylation and many phenotypes, but do not take into account information from neighboring probes. Methods to detect differentially methylated regions (DMRs) (clusters of neighboring probes associated with a phenotype) may provide more power to detect associations between DNA methylation and diseases or phenotypes of interest.

**Results:**

We proposed a novel approach, GlobalP, and perform comparisons with 3 methods—DMRcate, Bumphunter, and comb-p—to identify DMRs associated with log triglycerides (TGs) in real GAW20 data before and after fenofibrate treatment. We applied these methods to the summary statistics from an EWAS performed on the methylation data. Comb-p, DMRcate, and GlobalP detected very similar DMRs near the gene *CPT1A* on chromosome 11 in both the pre- and posttreatment data. In addition, GlobalP detected 2 DMRs before fenofibrate treatment in the genes *ETV6* and *ABCG1*. Bumphunter identified several DMRs on chromosomes 1 and 20, which did not overlap with DMRs detected by other methods.

**Conclusions:**

Our novel method detected the same DMR identified by two existing methods and detected two additional DMRs not identified by any of the existing methods we compared.

## Background

DNA methylation has been implicated in a number of diseases and is increasingly being used as target for drug therapies [1]. DNA methylation arrays allow for affordable epigenome-wide association studies (EWAS), but there is evidence that the standard single-probe approach is underpowered [2]. Cancer studies have found that hypermethylation of many probes in a gene promoter is associated with gene silencing, indicating that analyzing methylation by region may be more powerful [3]. There are several existing methods to detect differentially methylated regions (DMRs) by combining EWAS summary statistics [4–6]. Bumphunter smooths regression coefficients of neighboring probes to identify regions with higher coefficients than expected by chance. DMRcate implements a similar approach, smoothing and combining probe *t*-statistics to detect regions enriched for associations with a phenotype. Comb-p uses metaanalysis to combine the *p* values of neighboring probes to detect regions enriched for association.

We compared these 3 existing methods—DMRcate, Bumphunter, and comb-p—to a new algorithm, GlobalP, to detect DMRs using the real GAW20 data from the Genetics of Lipid Lowering Drugs and Diet Network (GOLDN) study.

## Methods

### Sample

GOLDN study participants were recruited from the Family Heart Study in Minneapolis, MN, and Salt Lake City, UT [7]. All participants were self-reported to be white. Triglycerides (TGs) were measured at 4 visits, and all participants were treated with 160 mg/day of fenofibrate after 3 weeks, between visits 2 and 3 [8].

DNA methylation in CD4+ T cells was measured using the Illumina Infinium Human Methylation 450 K BeadChip (Illumina 450 K) array before treatment at the second visit and after treatment at the fourth visit. The Illumina 450 K array measures DNA methylation at approximately 480,000 cytosine-phosphate-guanine (CpG) sites [9]. From this array, we obtain approximately 480,000 beta values between 0 and 1 for every participant, which represent the percentage of DNA strands that are methylated at each of these CpG sites, or probes. Beta values are easily interpretable, but often have severe heteroskedasticity. Therefore, some methods use methylation M values instead, defined as $M={\mathit{\log}}_2\left(\frac{\beta }{1-\beta}\right)$ [10]. There were 997 participants from 182 families with methylation data at 1 or 2 exams.

### Methylation preprocessing

Analysis of Illumina 450 K array data is susceptible to several technical challenges. The array uses 2 different technologies, Infinium I and Infinium II probes, to measure DNA methylation. Infinium II probes are not as accurate as Infinium I probes for beta values close to 0 or 1 [9]. Differences between the 2 probe types must be corrected to avoid spurious enrichment of associations in Infinium I probes [11].

Data made available were adjusted for probe type by fitting a second-order polynomial to all Infinium I and Infinium II probe pairs within 50 base pairs (bp) of one another. Because there were remaining systemic differences in the range of Infinium I and Infinium II probe beta values, we further adjusted for probe type using beta-mixture quantile dilation (BMIQ) to avoid Infinium I probe enrichment bias [11]. Finally, we removed probes with a polymorphic C, G, or single-base extension with minor allele frequency greater than 0.05 and cross-reactive probes, defined as any probe with at least 46 of 50 bp in common with a sequence elsewhere in the genome [12]. This left 430,298 probes for analysis.

### DMR methods

For each DMR method, we analyze the relationship between the natural log of TGs and methylation at visits 2 and 4, adjusting for age, sex, study center, and smoking status (current, former, or never smoker).

Bumphunter creates regions by combining all probes within a user-defined pairwise distance. Bumphunter uses a linear model for each probe to predict methylation M values from TG, adjusting for covariates, and smooths the effect size estimates of all probes within a region, ordered by chromosomal position. Potential bumps, or DMRs, are defined as the collection of smoothed effect sizes for any region with an effect size above a user-defined threshold. The statistical significance of the average height and area of each potential bump is calculated using a bootstrap approach and adjusted for multiple testing [5]. We perform 100 bootstrap replications using a maximum pairwise distance of 600 bp and use the 99th percentile of calculated effect size estimates as the threshold. Because Bumphunter does not account for family structure, we limit our analyses to an unrelated subset of 176 individuals in the pre- and posttreatment analyses for this method.

DMRcate, comb-p, and GlobalP use precomputed summary statistics as input. We perform an EWAS using a linear mixed-effects model, implemented with the *lmekin* function from the *coxme* R package, accounting for relatedness using a kinship matrix computed from known pedigree relationships. There are 993 individuals included in the pretreatment association analysis and 499 in the posttreatment analysis.

DMRcate applies Gaussian kernel weights to smooth EWAS z-statistics of all probes in a seed region and computes a region *p* value for the sum of weighted squared *t*-statistics using a Satterthwaite approximation [4]. We use the recommended bandwidth for this analysis, which collapses any 2 probes or regions within 1000 bp of one another into 1 region [4]. We also use the recommended definition of a seed region (at least 1 probe with a false discovery rate [FDR] < 0.05) and scaling factor, C = 2, to calculate the Gaussian kernel.

Using *p* values from any test of association between methylation and a phenotype, comb-p calculates autocorrelation between probes and uses this autocorrelation and neighboring *p* values to calculate Stouffer-Liptak-Kechris (SLK)-corrected *p* values for each probe. A peak-finding algorithm is used to identify regions with enriched SLK-corrected *p* values. The significance of each region is then determined by applying a Stouffer-Liptak correction to the original *p* values of all probes in the region. To correct for multiple testing, a Sidak correction, based on the number of possible regions of the same size, is applied to the Stouffer-Liptak *p* values [6]. Following the authors’ recommendation, we define regions in this analysis as all probes within 200 bp of another probe and only test for significant DMRs in regions with at least 1 unadjusted probe *p* value < 10^− 3^.

Finally, we develop our own algorithm, GlobalP. We calculate a z-score for each probe, $z=\frac{\beta }{SE\left(\beta \right)}$, from the *lmekin* analysis. It can be shown that under the null hypothesis of no association between methylation and TG and assuming that there are no covariates in the model, the vector of probe z-scores in a region with m probes, z_m_, follows a multivariate normal distribution with mean 0 and covariance Σ, where Σ is the *m* × *m* correlation matrix between probes in the study sample [13]. It follows from the properties of the multivariate normal distribution that ${z}_m^T{\Sigma}^{-1}{z}_m\sim {\chi}_m^2$. In the presence of covariates, Σ is the partial correlation matrix between probes [13].

Rather than grouping probes by a set distance, we group probes by annotation. We map probes to subcategories of genes and CpG islands, which are areas in the epigenome with more CpG sites than expected by chance [14]. We map probes using the UCSC definitions of gene annotation boundaries and CpG islands provided in the *IlluminaHumanMethylation450kanno.ilmn12.hg19* R package. There are 5 possible annotation categories within each CpG island: south shelf, south shore, island, north shelf, and north shore. CpG island shores are regions within 2 kb of a CpG island, and CpG island shelves are regions within 2 kb of a CpG island shore. For each gene, there are 6 “functional” annotations: the gene body; first exon; 5′ untranslated region (UTR); 3′ UTR; the region from the transcription start site (TSS) to 200 bp upstream; and the region from 200 bp to 1500 bp upstream of the TSS [9]. Of the probes we analyzed, 375,805 (87.3%) fall within at least one of these 178,015 annotations. For each annotation within a gene or CpG island, we calculate a *χ*^2^ statistic using the z-scores and partial correlation between probes. Because these annotations are not mutually exclusive, a Bonferroni correction is too conservative. We use an FDR cutoff of 0.05.

## Results

Tables 1 and 2 show the boundaries and significance of each DMR by method for TG levels before and after treatment, respectively. GlobalP, comb-p, and DMRcate identified at least 1 shared region. Comb-p identified only 1 DMR associated with both pre- and posttreatment TG in the 5′ UTR region of the gene *CPT1A* on chromosome 11. DMRcate identified this same region on chromosome 11 before treatment but not after. Although GlobalP identified 2 regions on chromosome 11, the 5′ UTR region of *CPT1A* and the north shore of a CpG island near *CPT1A*, both region signals were driven by the 2 probes they share in common, cg00574958 and cg17058475. In addition, GlobalP identified 2 DMRs, the body of the gene *ABCG1* and the body of the gene *ETV6*, not found using other methods.

**Table 1:** Genomic position (hg19) and significance of DMRs identified to be associated with TGs in pretreatment data

Chromosome	Start	Stop	No. probes	DMR *p* value	Method
1	247802703	247803688	7	< 0.01	Bumphunter
11	68583422	68609203	15	3.25E-14	GlobalP
11	68607622	68607676	2	1.01E-06	Comb-p
11	68607675	68607737	2	1.69E-21	DMRcate
11	68606155	68608154	3	1.30E-15	GlobalP
12	11803199	12038352	31	2.34E-3	GlobalP
20	443632	443641	2	3.00E-2	Bumphunter
21	43620345	249212500	23	2.34E-3	GlobalP

**Table 2:** Genomic position (hg19) and significance of DMRs identified to be associated with TGs in posttreatment data

Chromosome	Start	Stop	No. probes	DMR *p* value	Method
1	26880547	26881009	5	1.00E-02	Bumphunter
1	111742515	111744309	5	1.00E-02	Bumphunter
1	161183762	161185092	6	2.00E-02	Bumphunter
11	68583422	68609203	15	8.83E-11	GlobalP
11	68607622	68607676	2	6.84E-06	Comb-p
11	68608155	68609419	3	1.41E-12	GlobalP

**Fig. 1:**
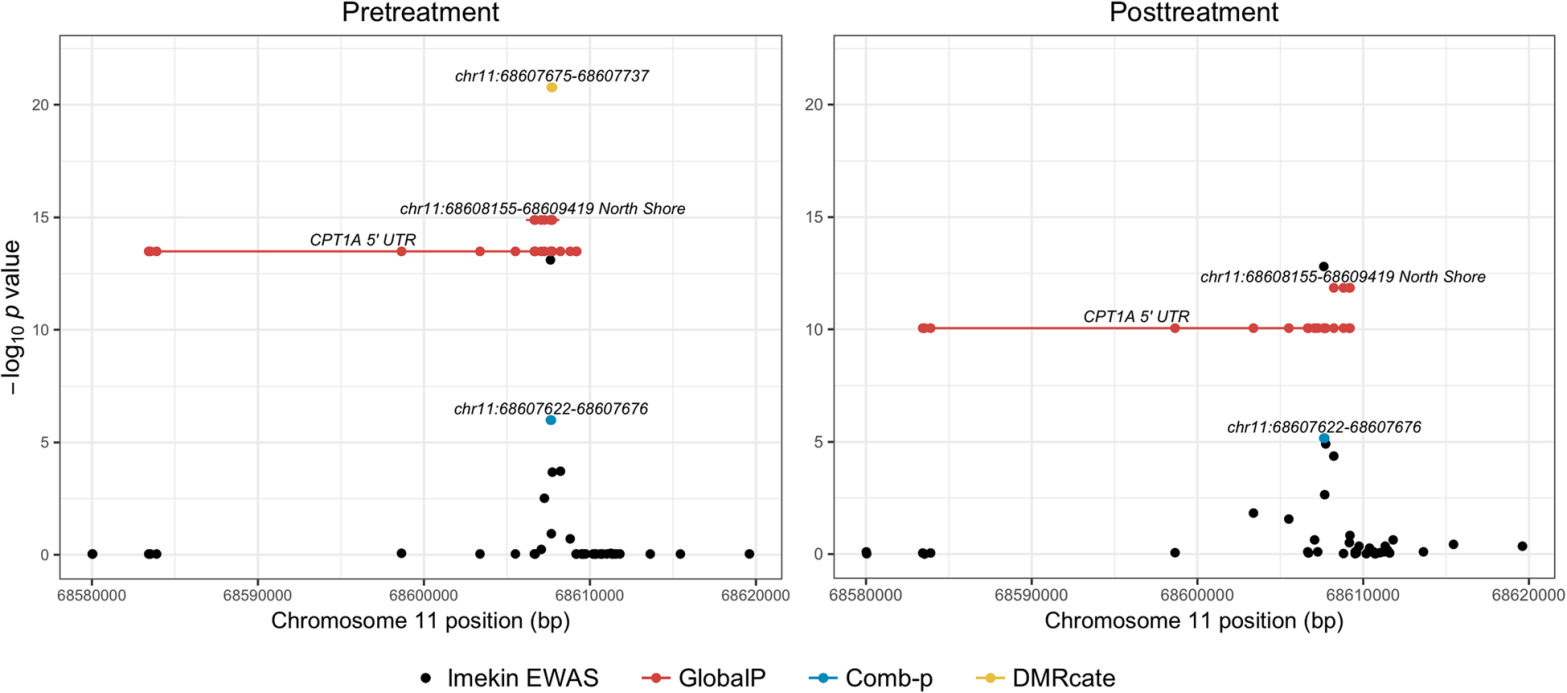
Analysis results (−log 10 *p* value) and position of DMRs before (*left panel*) and after (*right panel*) treatment in the *CPT1A* region on chromosome 11. Points represent single CpG site results from the *lmekin* EWAS and points connected by lines represent DMRs, with each point representing a CpG site in the region. DMRcate and comb-p DMRs are labeled with genomic position and GlobalP DMRs are labeled with gene or CpG island annotation. Comb-p *p* values were corrected for multiple testing using a Sidak correction, and all other *p* values were corrected for multiple testing using an FDR

Figure 1 shows the DMRs identified in the *CPT1A* region compared to the single-probe EWAS results in this region. At both time points, comb-p found a weaker association than the single-probe analysis found. However, in the pretreatment data, the aggregate signal in the region was stronger for DMRcate and GlobalP than the individual probe associations.

Bumphunter identified 2 regions in the pretreatment analysis, one on chromosome 1 and one on chromosome 20, and 3 regions in the posttreatment analysis, all on chromosome 1. The regions detected by Bumphunter did not overlap with regions identified by other methods.

To evaluate the Type I error of GlobalP, we analyzed the 200 simulated posttreatment GAW20 data sets (*N* = 717) using a 0.05 significance level, excluding the 5 simulated CpG sites associated with TGs. The average Type I error of both *lmekin* (mean error rate = 0.066) and GlobalP (mean error rate = 0.077) were slightly inflated.

## Discussion

One advantage to GlobalP is that by using predefined, fixed annotations, it is not sensitive to user input and can give meaningful probe categorizations. DMRcate and comb-p are flexible, but user choices may affect the validity of results. There is previous evidence that the 2 genes identified solely by GlobalP, *ABCG1* and *ETV6,* are associated with TGs and obesity, suggesting that these known biologic annotations can help identify biologically relevant associations [15, 16].

The Type I error rate of GlobalP was slightly inflated. While false-positive rates of all 3 existing methods have been demonstrated to be well controlled, we did not evaluate their Type I error rate in the current data set because of computational burden [4–6]. However, the original report on DMRcate included a warning that Type I error rate will increase if the probe FDR < 0.05 criterion for starting a seed region is relaxed. Therefore, it is impossible to detect aggregated weak signals (FDR > 0.05 for all probes) in a region without increasing Type I error [4].

GlobalP is less computationally intensive than the bootstrap approach of Bumphunter, but more computationally intensive than DMRcate and comb-p, largely because GlobalP uses individual data to calculate the partial correlation between each set of probes. It is common in gene and pathway analysis of genetic data to use an external reference sample to estimate correlation between genetic variants [17–19]. One future direction would be to perform a sensitivity analysis using precomputed probe correlations from publically available data, which would reduce the computational burden and allow for analysis of published EWAS results.

## Conclusions

We proposed a new method, GlobalP, to detect DMRs associated with TGs in both pre- and posttreatment methylation data from GAW20. We evaluated the Type I error rate of GlobalP, and compared it with three existing methods: comb-p, DMRcate, and Bumphunter. The Type I error rate of GlobalP was slightly inflated. This could potentially be caused by the inflated Type I error rate of the *lmekin* EWAS, but further investigation is needed to address this. DMRs identified by our novel method, GlobalP, were similar to the ones identified by comb-p and DMRcate, but did not overlap with the ones identified by Bumphunter. GlobalP was capable of detecting additional DMRs not identified by the other methods and found stronger evidence of association in the *CPT1A* region than comb-p found.

## Abbreviations

BMIQ: Beta-mixture quantile dilation
bp: Base pairs
CpG: Cytosine-phosphate-guanine
DMR: Differentially methylated region
EWAS: Epigenome-wide association study
FDR: False discovery rate
GOLDN: Genetics of Lipid Lowering Drugs and Diet Network
Illumina 450 K: Illumina Infinium Human Methylation 450 K BeadChip
SLK: Stouffer-Liptak-Kechris
TGs: Triglycerides
TSS: Transcription start site
UTR: Untranslated region
